# Ethical challenges in the treatment of psychotic pregnancy denial

**DOI:** 10.3389/fpsyt.2024.1337988

**Published:** 2024-02-02

**Authors:** Roshen John, Gabriel Tudose, Chin Kuo, Gabriella Arth, Sammi Wong

**Affiliations:** ^1^ Department of Psychiatry and Psychology, Mayo Clinic, Rochester, MN, United States; ^2^ Department of Psychiatry, SUNY Downstate, New York, NY, United States; ^3^ Department of Psychiatry, Maimonides Medical Center, New York, NY, United States; ^4^ Department of Psychiatry, Brookdale Hospital Medical Center, New York, NY, United States

**Keywords:** decision-making, autonomy, beneficence, capacity, pregnancy, psychosis, denial, maternal health

## Abstract

**Background:**

There is a paucity of literature regarding ethical strategies for treating pregnant people with psychosis. While not uncommon, psychotic pregnancy denial is a psychotic illness in which patients have the delusion that they are not pregnant. The authors provide a literature review regarding psychotic pregnancy denial, present an unpublished case and its questions and dilemmas, and offer recommendations for resolving the ethical challenges these cases raise.

**Case:**

A 26-year-old, single, unemployed woman of no fixed residence was admitted for suicidal ideation. She had a history of psychosis, had multiple ER visits and at least one previous hospitalization, had minimal contact with psychiatric outpatient clinics, and had been poorly compliant with treatment recommendations. She was discovered to be about 31 weeks pregnant in the emergency room. Ultrasound exams revealed no fetal anomalies. This was the patient’s second pregnancy; her previous pregnancy resulted in an abortion. Her sole psychotic symptom was the delusional belief that she was not pregnant. On the rare occasions when the patient acknowledged being pregnant, she requested termination of pregnancy. Despite intensive pharmacological treatment of her psychosis, the patient continued believing that she was not pregnant and repeatedly said she would not participate in the labor and delivery process. She disagreed with the induction of labor or a cesarean section if needed. The patient developed gestational hypertension, an obstetric indication for delivery. Induction of labor was offered to avoid potentially disastrous outcomes for the pregnant woman and the fetus.

**Conclusion:**

Psychotic pregnancy denial is potentially life-threatening. Delivery of the fetus requires carefully weighing risks and benefits and thoroughly considering the ethical framework.

**Teaching points:**

Treatment of birthing people with psychotic denial of pregnancy is complex; it requires special clinical and ethical skills to determine the patient’s level of decision-making impairment and to find a middle ground between the pregnant person’s right to autonomy and the physicians’ beneficence-based duties. Using a well-coordinated, interdisciplinary approach and a solid ethical framework, the decision to deliver the fetus while engaging the pregnant person, to the extent possible, in the decision-making process is essential.

## Introduction

Pregnant people with psychiatric conditions raise numerous clinical and ethical challenges. They are more likely to have unrecognized medical problems, poor self-care, unstable living situations, substance use, histories of sexual exploitation, and lack of supportive relationships. Psychotic illness, in particular, may undermine a patient’s ability to recognize labor and participate in recommended obstetric care. Patients with psychotic illness are less likely to receive prenatal care and more likely to present later in pregnancy; which leads to worse outcomes, including missed opportunities for prenatal diagnosis and treatment, intrauterine growth restriction, and lower Apgar scores (1–3). These patients have an increased need for interventions (i.e., amniotomy, pharmacologic induction or augmentation of labor, and emergency Cesarean section) (4, 5). Their postpartum experiences are often marked by psychotic relapse, parenting difficulties, and high rates of custody loss (6). While they often require intensive, multidisciplinary care, pregnant patients with psychosis are also more likely to lack access to such care.

Psychotic denial of pregnancy is a particular form of psychosis, not yet formally recognized as a distinct diagnostic entity, in which the pregnant person has the delusional belief that they are not pregnant. The incidence of denial of pregnancy at 20 weeks gestation is approximately one in 475, a rate similar to eclampsia, whereas the proportion of cases persisting until delivery is about one in 2,500 (7–9). People with psychotic pregnancy denial usually have a co-morbid psychiatric illness, most frequently schizophrenia, are often psychotic throughout the pregnancy, are usually unemployed and single, have been pregnant before, make no efforts to hide their pregnancy, and attribute fetal movements and other pregnancy symptoms to manifestations of the underlying psychosis (7). They are unlikely to comply with prenatal care and are at an increased risk of poor nutritional status, poor weight gain, exposure to teratogens, and fetal growth restriction (9). They typically present to the hospital in the advanced stages of pregnancy or even in active labor. Relatively little is known about the treatment and ethical challenges of this condition.

## Example case

A 26-year-old single, homeless, nulliparous woman presented to the emergency department reporting suicidal ideation. She had a history of psychosis, one past psychiatric hospitalization, and minimal contact with outpatient clinics. She was on no psychotropic medication at the time of admission. The patient was discovered to be about 31 weeks pregnant in the emergency room. The patient’s sole psychotic symptom was her delusion of not being pregnant. She disputed the significance of specific findings, such as pregnancy tests and ultrasound results, and did not accept the reality of her pregnancy. Furthermore, she substituted it with an alternative reality, stating that her distended abdomen was due to gas and having eaten rotten food. In the rare instances when the patient acknowledged her pregnancy, she claimed to be only seven weeks pregnant, requested an abortion, and stated that she “would not push anything out” and would “not be cut open”; this was further evidence that her decision-making capacity was impaired. The patient’s superficial level of engagement was due to her inability, rather than unwillingness, to engage in a meaningful decision-making process. The patient did not endorse any neurovegetative symptoms of depression or symptoms of mania or hypomania. While her suicidal thoughts subsided a few days after admission, the patient remained delusional. Early in her hospitalization, the patient had two episodes of unprovoked agitation, during which she attacked staff members. The patient was started on Haloperidol, which was gradually titrated up to 15 mg twice daily. She was compliant with her psychotropic medication but continued to refuse prenatal vitamins because she continued to think that she was not pregnant. While the patient was better related than before and her behavior was contained, she remained delusional. Obstetricians started following up on the case. The psychiatric team assumed the role of being the formal evaluator of capacity while allowing the obstetric team to build rapport over time, hoping this approach would improve the patient’s engagement and decision-making ability (10). The ultrasound revealed a viable fetus with no anomalies. In an attempt to maximize the therapeutic effect of the antipsychotic treatment and in the hope of restoring the patient’s capacity (11, 12), Olanzapine was added, and its dose was gradually titrated up to 40 mg daily. Given her history of documented medication noncompliance, the patient also received monthly intramuscular injections of Haloperidol decanoate 150 mg.

The patient requested a hearing for release, and her request was denied. She continued to take her psychotropic medication and started taking her prenatal vitamins, yet maintained that she was not pregnant. The team could not assess what she would want to do had she acknowledged she was pregnant. The team formally asked a judge to allow the clinicians to administer non-psychotropic medications and obstetric treatment over objection, given that she was deemed to lack capacity. However, while no ruling was in sight, the patient developed gestational hypertension, an obstetric indication for delivery to avoid potentially disastrous outcomes for the pregnant woman and the fetus (2, 4, 13). Once labor is induced, vaginal delivery would ensue, but in some instances, due to obstetric reasons, a Cesarean section might be needed. If the delivery would be vaginal, the concern was that the pregnant patient would not comply with the clinicians’ instructions to ensure the safe delivery of the infant. If a Cesarean section would be required, the clinicians struggled with the fact that even if a patient who lacks capacity declined a surgical procedure, they would not want to perform a Cesarean delivery.

A decision was needed concerning the method of delivery. Prompt delivery was required to avoid maternal and fetal harm. Since the patient's family members declined involvement, the physicians identified the patient's male friend as the surrogate decision-maker. The friend was the only person who visited the patient during her hospital stay, was interested in her well-being, and the patient wanted him to be involved in the process. Multiple learning trials, repetition, simplification, and organization of information in language at the patient’s comprehension level are vital in obstetricians’ treatment plans to attempt to reduce the patient’s understanding deficits. Physicians explained to the patient and to the surrogate the indications, the risks and benefits of the procedures, the alternatives, and the risks of non-intervention. The patient could absorb, retain, and recall information provided to her. The team informed the patient that every attempt at vaginal delivery would be made but that a Cesarean section would be resorted to should there be a determination of risk to her or the fetus. The patient assented verbally to participate in labor and delivery with the understanding that Cesarean section might be unavoidable, and both she and the surrogate signed the consent to proceed with the induction (14, 15). The patient’s labor was induced, but the induction failed. Consequently, a Cesarean section was required. The patient had an uncomplicated Cesarean procedure at 38 weeks gestation, delivering a healthy female infant. The infant and the mother were briefly separated at birth due to concerns that the mother might harm the infant. After preparing the patient to see the baby, they spent some time together under careful supervision.

The patient regained capacity after delivery, no longer expressing delusional beliefs, expressing an interest in her infant, and acknowledging the infant’s existence. The obstetrics team discussed with the patient her choices and preferences about future pregnancies; the patient chose to receive a long-acting injectable contraceptive. A custody hearing occurred the day after the patient was discharged. The patient requested custody but the infant was placed in foster care.

## Discussion

### Is the patient capable of making medical decisions?

One of the first steps in treating a patient with psychotic denial of pregnancy is establishing if the patient has decision-making capacity. The patient’s psychotic symptoms do not, unto themselves, prove a lack of capacity. Psychiatrists are often first called to evaluate a patient’s decision-making capacity when the patient’s decision is misaligned with the clinical team’s recommendations (16).

One question is whether the patient is unable or unwilling to recognize facts that are perceptually obvious to others? (17). In our case, the patient disputed the significance of pregnancy tests and ultrasound results and offered illogical explanations for her distended abdomen. The other question is whether the patient’s impaired decision capacity is driven by her psychotic illness, or it is a choice that others might make in similar circumstances. For instance, had the patient justified her decision not to give birth by the fact that the fetus had a severe congenital malformation or was the carrier of a lethal gene that would significantly reduce its life expectancy, that decision, while not necessarily condoned by the clinicians, would have been understandable (15). However, when the patient challenges the mere essence of her pregnancy, psychosis is the factor that drives her to make such a decision.

Clinicians need to clarify how reversible the underlying condition causing the decision-making impairment is. For example, the literature mentions cases of pregnant people with major depression and psychosis who, after receiving adequate psychiatric treatment, would regain their decision-making capacity while still pregnant (18). On the other hand, it is doubtful for pregnant patients in a coma to regain capacity.

Patients with psychotic denial of pregnancy often vacillate between categorically denying their pregnancy and superficially acknowledging it. This highlights the importance of serial capacity assessments to accurately determine a patient’s ability to make medical decisions over time.

Assessing a pregnant patient’s capacity is ethically challenging due to the “high stakes” decisions involved, the multitude of stakeholders— the pregnant patient, clinicians, the father of the infant-to-be, the patient’s family, the hospital, committee panels, the state, to name a few—, and the often irreversible and time-sensitive nature of these decisions such as abortion, the time and mode of delivery (19).

Reconciling the patient’s right to autonomy with the clinician’s beneficence-based obligations is particularly challenging. The default locus of decision-making for the fetus should be with the pregnant person; the patient is considered best able to evaluate the mix of risks and benefits from a maternal and fetal perspective (20). However, a pregnant person’s refusal of any obstetric treatment brings the principles of autonomy and beneficence into conflict. Are ethical principles fixed? Can one ethical principle prevail over the other? Is it possible to preserve the patient’s autonomy while protecting fetal well-being? These dilemmas arise when patient autonomy and physicians’ beneficence-based decisions are misaligned (20).

One could argue that “high stakes” decisions, such as labor induction and delivery time and mode, should require a higher capacity threshold. That is, the higher the risks associated with the treatment decision, the greater the capacity required to make the decision. Some advocate for substantive risk-sensitivity (i.e., higher risk raises the threshold for capacity), while others favor epistemic risk-sensitivity (i.e., higher risk raises the requirement for evidence of intact capacity). The main objection to the “sliding scale” approach is that it shifts the onus onto the patient to prove capacity when, in fact, it is the psychiatrist who needs to show incapacity through more intensive, serial capacity assessments. Nevertheless, the “sliding scale” approach implies that decision-making capacity is far from a binary concept—patients have varying degrees of impairment, from intact capacity to reduced (or impaired) capacity to no capacity (21). Determining the threshold for decision-making capacity implies a balancing act between giving patients as much freedom as possible and preserving their welfare. In our case, the patient could absorb, retain, and recall information provided to her. However, her disavowal and substitution of reality left her unable to appreciate future likely consequences (impaired cognitive understanding) (2, 22). Our patient’s decision was based on her delusion that she was not pregnant rather than on her assessment of the risks of such interventions. In addition, the patient lacked appreciation since she did not understand the consequences of her decision on her and her fetus.

Clinical challenges also complicate the treatment of pregnant people. Clinicians’ countertransference or professional and personal commitments should not influence the physicians’ assessment of the patient’s decision-making capacity (16). It is easy to understand, for instance, how a pregnant person who is angry and attacks staff or is poorly engaged generates a strong negative emotional reaction that would bias the physicians’ assessment of her capacity (2). Similarly, clinicians’ risk-aversion, professional fears, and staff’s political or religious views could cloud the clinical judgment and introduce bias in assessing the patient’s capacity. Being entirely objective about a pregnant person’s capacity is a challenging task. A system of checks and balances in the form of second-opinion evaluations, interdisciplinary discussions, and informal conversations with colleagues should be in place to prevent clinicians from imposing their values as a standard for decision-making.

A patient’s poor engagement may create conflicts between the patient and the treating team as it would not allow physicians to explore the patient’s values and preferences (10). The pitfall is that clinicians tend to focus on history, mental status exam, and collateral information, without exploring the patient’s choices.

### Who decides?

Pregnant patients’ impaired decision-making capacity should not be used against them to infringe on their rights to autonomy and bodily integrity (23, 24). Physicians need to identify any reversible barriers to the patient’s exercise of autonomy. Therefore, educating patients about the process of labor and delivery and about the way psychiatric illness might contribute to their difficulties with decision-making is an essential part of the shared decision-making process that respects patients’ autonomy (20, 25). Using an empathetic stance and language to the best of the patient’s understanding may help them better comprehend their choices (25). Multiple learning trials (repetition) might reverse some barriers to the patient’s understanding. Pharmacological interventions can benefit patients with impaired capacity secondary to depression; less is known about the benefits of psychotropic medication in restoring capacity to patients with psychosis.

A surrogate should be identified when a patient’s capacity is impaired. (25). A surrogate is usually someone who knows the patient well and is invested in her well-being, e.g., the spouse, a family member, or a close friend. One should take into account the fact that several people might identify themselves as potential surrogates and that they might have conflicting opinions regarding the patient’s care. Similarly, one could conceive of situations when no surrogate can be identified. Our case did not fall into either category as only one person, a male friend acceptable to the patient, visited the patient and expressed interest in her well-being (2).

### How are decisions made?

In the sliding-scale paradigm, patients with low capacity need directive guidance and a surrogate, whereas patients with medium decisional capacity require a mixed decision approach and a surrogate. Surrogate decision-maker’s engagement becomes progressively more indispensable as decision-making capacity wanes. Using the shared decision-making approach is meant to preserve the patient’s autonomy; it allows the physicians and the surrogate decision-maker to make decisions with, and not for, the patient, thus avoiding the slippery slope to paternalism (20, 25). Defining the degree of impairment that constitutes a lack of capacity should reflect society’s view of the appropriate balance between patient autonomy and protecting and promoting the patient’s well-being. Consequently, the rigorousness of the competence assessments correlates directly with the seriousness of the likely consequences of patient’s decisions. Although this sliding scale approach raises objections, it makes sense from a policy standpoint (21). Physicians must be cautious not to allow their determination of the patient’s capacity (or lack thereof) be influenced by whether or not the patient’s choices and decisions align with clinical recommendations (16).

The surrogate and the physicians should protect maternal well-being and avoid fetal harm. The surrogate decision-maker will make, to the extent it is possible, a decision based on the patient’s values—the decision the patient would make if the patient were capable of doing so (substituted judgment standard) (3, 8, 25). In our patient’s case, the surrogate decision-maker did not know what the patient would have decided regarding her pregnancy and delivery.

If the patient’s wishes are unknown, the surrogate decision-making should be guided by the best interests standard, i.e., identifying the treatment that protects and promotes the patient’s health (2, 3). Physicians need to differentiate between their expectations of proper patient behavior, a life worth living for, and treatment of choice on one hand, and patient values on the other.

The fluidity of intrapartum decision-making raises several challenges for applying the best interest standard. Complex matters require time and reflection, yet given the time-sensitive nature of obstetric events, there is a high likelihood they could be managed poorly. Nevertheless, neither the time constraints of obstetric procedures nor the unpredictability of psychiatric symptoms should be an excuse for not engaging a patient in decision-making. Pregnant people may rapidly face a series of complex, anguishing decisions, most of them time-sensitive. In our case, the patient developed gestational hypertension, an indication for induction of labor and, if needed, a Cesarean section (2, 4).

The patient’s firm refusal of Cesarean section creates a dilemma. Both forced maternal treatment and physicians’ non-intervention leading to the injury or death of the fetus or infant have adverse consequences. On one hand, forced maternal treatment weakens the liberty of pregnant people, leads to the traumatization of the treaters involved, and disrupts the physician-patient relationship. On the other hand, non-intervening and allowing the fetus or infant to die has a similar traumatizing effect on clinicians, weakens respect for life, affects therapeutic relationships, and patients might become less secure in their belief that physicians do not kill one patient for the sake of another. Ultimately, the pregnant person’s capacity (or lack thereof) tilts the balance one way or another and determines whether the patient’s autonomy or the clinicians’ beneficence-based obligations prevail (12).

Weighing the risks and benefits in pregnant people who lack capacity involves a complex judgment. While literature shows that psychotic denial of pregnancy is associated with infanticide, this risk is difficult to predict. Unknown risks, by definition, cannot be dismissed as being minimal (20, 24). Unattended precipitous labor and delivery is another potentially life-threatening risk to the patient and the neonate. The denial of the inevitable can lead to disastrous consequences.

### Whose best interest?

A fundamental ethical question is, who exactly is the physician’s patient? While everyone agrees that the pregnant person is the patient, many argue that the fetus is also a patient (2, 7, 14). Both ethically and legally, the fetus’s personhood is debatable. There is no clear definition that a patient should be a person. On one hand, one could view the fetus as a “parasite” or a “growth” within the pregnant person’s body (26). Any attempt to treat or remove the “growth” would violate the patient’s bodily integrity and autonomy, at least as long as the patient can make medical decisions (23). On the other hand, the fetus, as the sole possessor of specific conditions the pregnant patient does not have, could be viewed as a patient residing in the pregnant person’s body. In either model, it is wrong to allow physicians (or the state) to subsume their interests and the civil rights of pregnant patients to those of the fetuses within them. Formulating the fetus as a patient does not imply that the fetus is a person or a separate entity. This concept implies that the fetus is a patient inside the pregnant person’s body and that physicians, as fiduciaries of their patients, have an obligation toward the fetus as well (15). Furthermore, this establishes a complex and asymmetrical relationship between the physician and the two intertwined patients; the physician does not have equal duties toward the two patients since the fetus’s treatment will inevitably require it to go through the pregnant person’s body. The demarcation between pre-viable and viable fetuses around the 24th week of gestation is not compelling (2). While the obligation to the fetus increases in more advanced stages of pregnancy, it never supersedes the pregnant person’s right to negative autonomy (e.g., the right to refuse a Cesarean section, even if the consequence of that refusal is fetal demise) (15, 23).

Pregnant people cannot be held to frivolous or unproven standards in the name of fetal welfare or be held to different standards than non-pregnant patients. Pregnant patients can also make poor choices and show poor judgment (i.e., use of alcohol or drugs during pregnancy), with clear adverse outcomes on their pregnancies; this does not give physicians (or the state) license to violate pregnant people’s autonomy (27). Nevertheless, when physician non-intervention might lead to the pregnant person’s and/or fetus’s death, or when the patient is not able to make decisions, the person’s autonomy and right to bodily integrity come into conflict with the clinicians’ beneficence-based duties (16). While the four pillars of medical ethics (autonomy, beneficence, non-maleficence, and justice) are, in theory, equal, some view autonomy as the “first among equals” (25). Do physicians have an obligation to the fetus to ensure its well-being that cannot be abrogated by the parent (28)? Can these obligations supersede the pregnant person’s autonomy? Is it possible to reconcile respect for the pregnant person’s autonomy with the protection of fetal well-being? These are dilemmas that arise when patient autonomy and physicians’ beneficence-based decisions are misaligned (16).

The pregnant person’s role as a moral fiduciary of the fetus is ethically complex (2). For late gestational-age fetuses, pregnant people (and physicians alike) have beneficence-based moral obligations toward the fetus (14, 15). When pregnant patients, through their incapacity to make decisions, put themselves or the fetus’s safety in imminent danger, clinicians need to weigh two distinct and conflicting values against each other. Would autonomy or beneficence prevail (12)? Had autonomy and free choice been society’s sole value, physicians would not need to assess capacity, and they would follow patients’ decisions and honor their requests. That this is not the case, that autonomy is not absolute and that patients’ welfare is also crucial is proven by the fact that clinicians do not mindlessly follow patients’ wishes. Nevertheless, the ethical dilemma between autonomy and beneficence cannot be reduced to an either/or binary. As the patient’s mental capacities are increasingly more compromised, the risks to welfare posed by free choice increase, and so does the need to protect the patient’s well-being. Had the fetus had a significant anomaly incompatible with survival, the ethical interest in promoting a favorable fetal outcome would have carried little weight against the desire to respect the person’s wishes and maximize her well-being. However, the case of our patient justifies some respect for the fetus. In this situation, respect for autonomy is not without limits.

### Judicial hearing vs ethics consult

Law is binary; ethics are not. Obstetrics deals with urgencies and emergencies. While the law is clear regarding the treatment of emergencies, it becomes murkier when treating urgencies (i.e., gestational hypertension, post-term pregnancies). Fears of litigation or of being criticized in case of a negative outcome make physicians inclined to resort to court hearings to address the treatment of pregnant people who lack capacity. However, given the adversarial nature of the legal system and the lengthy duration of the hearings, resorting to judicial hearings, irrespective of their rulings, is an undesirable venue to address this issue. Taking the patient to court has the potential of making adversaries out of the pregnant people; this would likely result in more harm than good because many patients will avoid physicians altogether during pregnancy if failure to comply with medical recommendations results in forced treatment or involuntary confinement. The concept of forcing a patient to undergo any procedure against their will, even if approved by a court, is morally reprehensible and ethically wrong and turns pregnant people into “fetal containers” (15). Court-ordered forced maternal treatments are rarely, if ever, of value in real-time patient care situations.

The alternative of a routine ethics consult is a better process to encourage consensus between the clinical team and the pregnant person in the decision-making process. It does not reduce the patient to a “fetal container” simply because other interests are recognized (2). In our patient’s case, the reason for the patient’s refusal was psychotic, and we chose to optimize her psychopharmacological treatment with the hopes of improving her ability to make a decision. The team informed the patient that every attempt at vaginal delivery would be made but that a Cesarean section would be resorted to should there be a determination of risk to her or the fetus. The pregnant patient would thus need to assent to the cesarean section as an acceptable option before induction of labor. Assent is possible when the patient retains enough of the components of decision-making capacity to express her values and preferences so physicians can make decisions with (and not for) the patient via the surrogate decision-maker (2, 14). Regardless of the decisions made, the patient still must be able to participate in labor and delivery.

### Preventive ethics

If the patient regains capacity, clinicians should engage the patient in formulating a plan to guide delivery decision-making if the patient’s capacity becomes impaired again (preventive ethics) (2). Physicians should discuss in advance that events during pregnancy can quickly place the patient or fetus at risk, and that psychiatric symptoms can unpredictably worsen to the point of the patient not having capacity while eliciting the patient’s point of view about Cesarean delivery for maternal or fetal indications. This strategy will help preserve the patient’s autonomy and respect their wishes should they someday lack capacity. In our case, the patient regained capacity after delivery, and the obstetrics team discussed with the patient her future pregnancies and preferences; the patient opted to receive a long-acting injectable contraceptive. Strategies of assisted decision-making when the patient has some capacity to protect patients with psychosis and chronically and variably impaired autonomy while preventing the clinicians from taking over the patients’ decision-making (2, 14, 22).

## Recommendations

Guiding pregnant people through the process of decision-making regarding their treatment can encounter conflicting interests between the patient, family members, and clinicians. However, there are no formal recommendations to help physicians navigate the complex ethical framework of treating patients with psychotic pregnancy denial so clinicians can reconcile preserving the pregnant person’s autonomy with clinicians’ beneficence-based duties. We make the following recommendations:

- Thorough serial capacity assessments by physicians with a particular set of clinical and ethical skills, second opinions, and serial assessments done by multiple people.
- Considering the patients’ needs above all, acknowledging the loss of autonomy associated with severe illness, and protecting patients who cannot make autonomous decisions.
- Training physicians in identifying and modifying their implicit biases (i.e., underlying emotions and thought patterns) to reduce the likelihood that personal distortions interfere with clinical decisions (16).
- Intensive interdisciplinary collaboration between Obstetrics, Psychiatry, and Ethics (19).
- Implementing preventive ethics when the patient regains capacity (22).
- Routine ethics consults rather than judicial hearings should be considered (20).
- Larger studies are needed to identify and characterize systematically those for whom induction of labor has been attempted for psychiatric reasons to provide future guidelines for the psychiatric consultant (4, 24).
- Identifying pregnant people at higher risk for worsening psychiatric symptoms to allow early intervention and close monitoring.

## Author contributions

RJ: Conceptualization, Investigation, Project administration, Resources, Writing – original draft, Writing – review & editing. GT: Conceptualization, Investigation, Project administration, Resources, Supervision, Writing – original draft, Writing – review & editing. CK: Conceptualization, Resources, Writing – original draft. GA: Conceptualization, Resources, Writing – original draft. SW: Conceptualization, Resources, Writing – original draft.
